# Legend of the Sentinels: Development of Lung Resident Memory T Cells and Their Roles in Diseases

**DOI:** 10.3389/fimmu.2020.624411

**Published:** 2021-02-02

**Authors:** Youkun Qian, Yicheng Zhu, Yangyang Li, Bin Li

**Affiliations:** Department of Immunology and Microbiology, Shanghai Institute of Immunology, Shanghai Jiao Tong University School of Medicine, Shanghai, China

**Keywords:** tissue-resident memory T cells, lung, infection, asthma, cancer, vaccine

## Abstract

SARS-CoV-2 is wreaking havoc around the world. To get the world back on track, hundreds of vaccines are under development. A deeper understanding of how the immune system responds to SARS-CoV-2 re-infection will certainly help. Studies have highlighted various aspects of T cell response in resolving acute infection and preventing re-infections. Lung resident memory T (T_RM_) cells are sentinels in the secondary immune response. They are mostly differentiated from effector T cells, construct specific niches and stay permanently in lung tissues. If the infection recurs, locally activated lung T_RM_ cells can elicit rapid immune response against invading pathogens. In addition, they can significantly limit tumor growth or lead to pathologic immune responses. Vaccines targeting T_RM_ cells are under development, with the hope to induce stable and highly reactive lung T_RM_ cells through mucosal administration or “prime-and-pull” strategy. In this review, we will summarize recent advances in lung T_RM_ cell generation and maintenance, explore their roles in different diseases and discuss how these cells may guide the development of future vaccines targeting infectious disease, cancer, and pathologic immune response.

## Introduction

The COVID-19 pandemic is ravaging the world. By the end of November 2020, there are over 60 million cumulative cases globally, and the number of deaths has exceeded one million (1). This disease is caused by SARS-CoV-2, which is mainly transmitted through air-borne droplets, leading to severe pulmonary diseases and systemic damage (2). Up to now, the treatment for COVID-19 is very limited, and no specific antiviral drug has been developed. Multiple candidate COVID-19 vaccines are undergoing clinical trials (3).

In general, most COVID-19 vaccines in clinical trials focus on humoral immunity, which exerts antibodies to prevent the virus from invading cells. However, antibodies alone may not be sufficient to prevent SARS-CoV-2 infection. One reason is that extracellular antibodies cannot completely clear the cells infected by virus (4). The final elimination of the virus depends on the supplement of cellular immunity, that is, the role of T cells, which help B cells produce neutralizing antibodies and can directly kill virus-infected cells. The second is that the memory B cell response tends to be short-lived (5), whereas the T cell response can last for many years. Recent researches have demonstrated that patients who recovered from the severe acute respiratory syndrome (SARS) still had long-lasting memory T-cells but reduced antibody responses (6, 7). Therefore, vaccines against SARS-CoV-2 should focus on activating the adaptive branch of the immune system and explicitly focus on inducing long-term memory T cells. Given that many respiratory viruses are controlled by tissue immune cells that may not be present in the blood, the tissue-resident memory T (T_RM_) cells infiltrated in the lungs that can recognize foreign antigens locally and provide a rapid immune response will be an area of concern.

Actually, CD8+T cells retained for a long time after influenza virus infection were observed in mouse lungs as early as 2001 (8). Extensive studies in mouse models have determined that the lungs are enriched in T_RM_ cells against a variety of viral and bacterial antigens brought by respiratory infections or vaccination. Specific T_RM_ cells were also detected in the respiratory tract of patients with influenza or tuberculosis (TB) (9). These pathogen-specific T_RM_ cells produced by prior exposure can control acute re-infections and achieve long-term immunity. In mouse model, an intranasal recombinant vaccinia virus boosting regimen has generated SARS-CoV-specific lung resident memory CD8+T cells. When re-stimulated, these T_RM_ cells can effectively release a variety of effector cytokines and cytotoxic molecules that prevent extensive virus replication and limit the alveolar damage (10). Another study suggested that the administration of SARS vaccine intranasally induced CD4+ T_RM_ cells in the respiratory tract of mice, which offered the protective immunity against death (11). Regarding SARS-CoV-2, recent published single-cell profiles have indicated that the CD8+ T cells in bronchoalveolar lavage fluids (BALFs) of patients with severe infection exhibited a less proportion of tissue-resident phenotypes than those in moderately infected patients (12). Hence a vaccine that induces the production of lung T_RM_ cells is an ideal candidate for generating a strong and rapid immune response against SARS-CoV-2.

There are other T_RM_ cells in the lungs with different roles, including T_RM_ cells that may cause pathological immune responses and tumor-infiltrating T_RM_ cells that can enhance anti-tumor immunity in the lungs (13). These T_RM_ cells under different immune microenvironment in the lungs act in various roles in immune defense, immune homeostasis, and immune surveillance. An in-depth understanding of the generation and maintenance of lung T_RM_ cells will provide new insights for the development of novel vaccine formation and delivery strategies and lung-specific immunoregulatory therapy.

This review will focus on the definition, generation, and different roles of lung T_RM_ cells in infection, pathological immune responses, and cancers, and discuss T_RM_ cell-related vaccination strategies combined with emerging cutting-edge discoveries.

## Hallmarks of T_RM_ Cells

T_RM_ cells, also known as non-circulating memory T cells, include both CD8+ and CD4+ subgroups. It refers to those memory T cells that occupy long-term residency in local tissues such as lung, intestine, and skin. Through cell labeling, parabiosis, tissue transplantation, and other methods, the circulation trajectory of cells can be observed to determine T_RM_ cells (14–16). However, it is still a challenge to clearly distinguish T_RM_ cells from other cells *in vitro* by surface markers.

In recent years, with the development of transcriptomics, T_RM_ cells have been found to have unique transcriptional profiles and functional characteristics. The main hallmarks of T_RM_ cells that distinguish it from other circulating memory T cells are the ability to adhere to peripheral tissues and the lack of homing signals. Based on the research on both mouse and humans, the most used phenotypic marker defining T_RM_ cell subsets is CD69. Due to the competitive protein-protein interaction between CD69 and sphingosine-1-P receptors (S1PR), it inhibits the expression of S1PR and prevents S1P-mediated egress (17, 18). These cells also lack CD62L and CC-chemokine receptor 7 (CCR7), both of which direct cells into lymphoid tissue (19). On the flip side, CD44 up-regulated by T_RM_ cells is the receptor for hyaluronic acid and other ligands expressed in peripheral tissues, which can induce the retention of memory T cells in peripheral tissues (20). As another key T_RM_ cell marker, the integrin αE:β7 (CD103) is mainly expressed on CD8+ T_RM_ cells and some on CD4+ T_RM_ cells, which binds E-cadherin and anchors cells around epithelial cells (21). It is worth noting that T_RM_ cells in lungs can be defined by several major surface markers, but this subset itself is still heterogeneous in some way. The transcriptome analysis reveals the inconsistent changes in gene expression among different cells (19, 22, 23). Further elucidation of detailed mechanism of T_RM_ cell formation and maintenance will add to understanding of the phenotype of lung T_RM_ cells under different pathophysiological conditions.

## Development of Lung T_RM_ Cells

**Figure 1:**
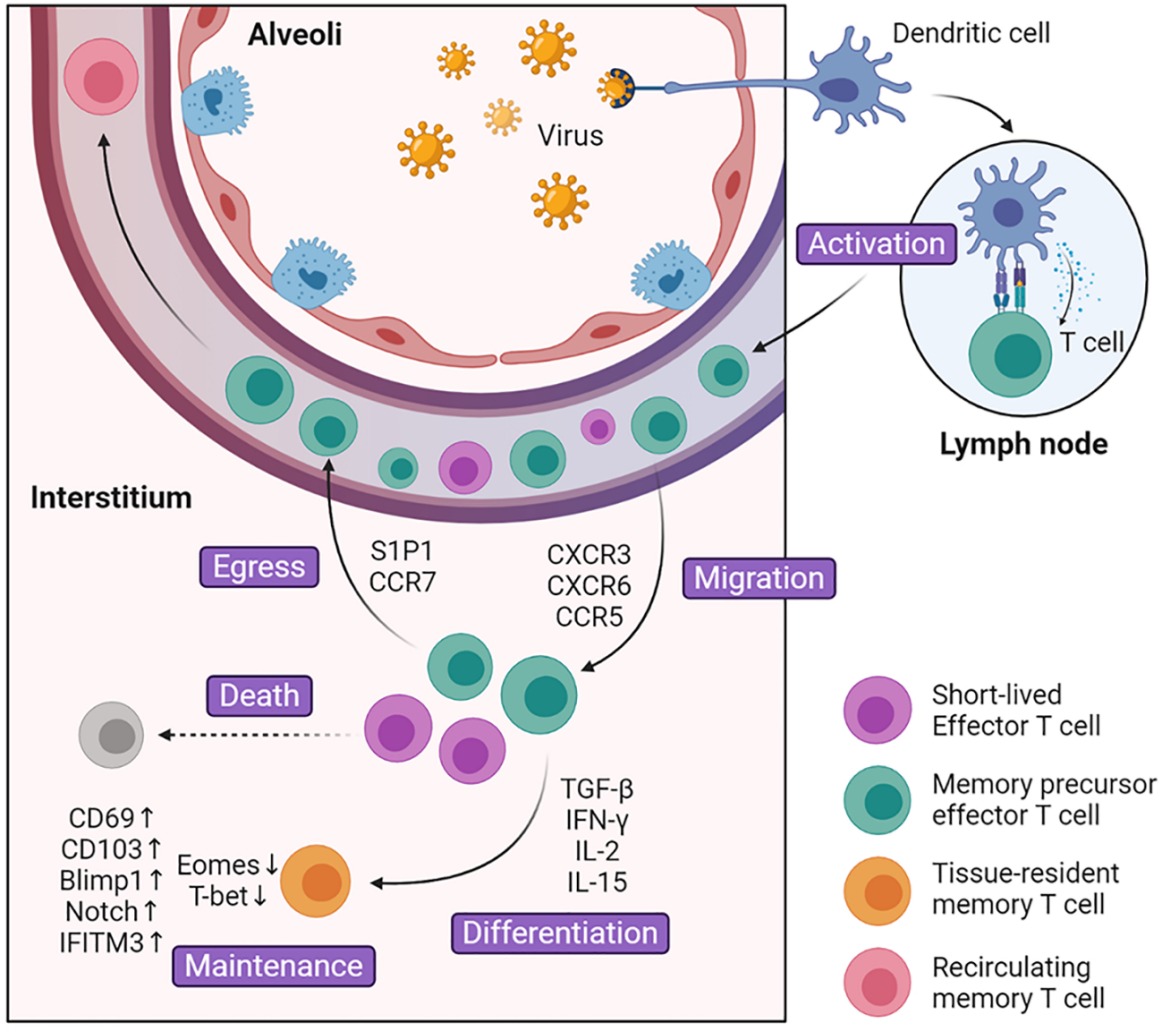
Generation and maintenance of lung T_RM_ cells. During the activated phase of infection, dendritic cells present antigens to activate naïve T cells in the lymph nodes. These cells turn into effector T cells and up-regulate surface marker CXCR3, CXCR6, CCR5, which guide them into inflammatory tissues. After entering lung tissue, part of effector T cells is regulated by environmental signals including cytokines such as TGF-β and cognate antigens, and differentiate into lung T_RM_ cells. The rest of the effector T cells undergoes cell death or egress out of the lung. Compared with Teff cells, lung T_RM_ cells manipulate multiple surface markers and transcription factors that facilitate cell maintenance and survival.

The development of lung T_RM_ cells can be divided into several steps: 1) activation in lymphoid tissues and migration into inflammatory lung tissue guided by local cytokines, 2) expression of homing molecules and specific transcription factors and differentiation into lung resident memory T cells, 3) local maintenance in specific niches and replenishment from T_CM_ cells (**Figure 1**). So far, the focus on specific transcription factors and cell surface receptors has gradually revealed details in the fate determination mechanism of lung T_RM_ cells.

### Activation and Migration

The inability to recirculate between lung and lymph nodes or bloodstream is a key determinant of lung T_RM_ cells (24, 25). However, these cells did not start in the lung tissue but migrated into it later. Under normal conditions, naïve T cells consecutively circulate throughout the body. When infection occurs, dendritic cells (DCs) migrate from infected respiratory sites into mediastinal lymph nodes (MdLN) and activate naïve T cells. Among these migrant DCs there are two subsets, and only airway localized CD103+ DCs can fully induce the differentiation of naïve T cells into T_eff_ cells (26). Once activated, the T_eff_ cells up-regulate the expression of CXCR3, CCR5, and CCR4, which specifically guide T_eff_ cells into lung tissue and help control pathogen invasion (27–31). For example, after TB infection, chemokine ligand IP-10 in the lung increases significantly, which binds to CXCR3 and facilitates T cell migration (29). In addition, CD8+ and CD4+ lung T_eff_ cells are regulated differently and tend to localize in different regions. CD8+ T_eff_ cells are inclined to migrate to the collagen IV-rich region and CD4+ T_eff_ cells are more prone to be located in areas abundant in collagen I (32). Compared with CD8+ T cells, CD4+ T cells enter the lung tissues first and direct the localization of CD8+ T cells. CD4+ T cells fine-tune chemokine gradients in the microenvironment such as TGF-β, which promotes the production of CD103 and is crucial for CD8+ T_RM_ cell formation (33).

### Differentiation

T_eff_ cells will not transform into lung T_RM_ cells immediately after entering the lung tissues. The tissue microenvironment has an important influence on the development of lung T_RM_ cells. In the early stage of infection, T_eff_ cells that migrate into the infection site will encounter redundant inflammatory signals, which guide T_eff_ cells towards terminal T_eff_ cells (34). They reduce local inflammation, help remold the microenvironment and make it more appropriate for the differentiation of lung T_RM_ cells. In the later stage, CD8+ T cells are recruited into tissue damage sites, which later developed into regenerative tissues termed as repair-associated memory depots (RAMDs). RAMDs provide environmental cues that help drive CD8+ T_eff_ cells into CD8+ T_RM_ cells and later become niches for CD8+ T_RM_ cells (35, 36). Predominant environmental cues include cytokines such as TGF-β, IL-33, TNF, IFN-γ, IL-15, and cognate antigens (18, 33, 37). TGF-β plays an important role in promoting the expression of T_RM_ cell marker CD103 and CD69. Together with IL-33 and TNF, TGF-β can provoke KLF2 down-regulation, which further down-regulates its target protein S1P1 and increases expression of CD69 (18). Furthermore, TGF-β down-regulate T-box transcriptional factor and promote the expression of CD103. T-box transcriptional factors are composed of eomesodermin (Eomes) and T-bet, and they vary in the degree of decline. While Eomes is effectively removed, T_RM_ cells maintain residual levels of T-bet which is important for T_RM_ cell survival (37). The decrease in production of T-box transcriptional factor is demonstrated in mature lung CD8+CD103+ T_RM_ cells (33, 37). Unlike CD8+ T_RM_ cells in other tissues like skin and vagina, where they can be generated with only local inflammatory signals (38), lung CD8+ T_RM_ cells must interact with cognate antigen before differentiation. After the exposure to cognate antigen, CD8+ T_eff_ cells increase the expression of CD69, CD103, and collagen-binding integrin VLA-1 (39). T cell receptor (TCR) signaling can also induce Blimp-1 expression, which biased CD8+ T_eff_ cell differentiation towards T_RM_ cells rather than T_CM_ cells (40). It is surprising that pulmonary monocytes and type 1 regulatory T (T_reg_) cells also contribute to the differentiation. Pulmonary monocytes are the major cells to present pathogen antigens, while type 1 T_reg_ cells promote the bioavailability of TGF-β (41, 42). As mentioned above, CD4+ T_RM_ cells have different development pathways compared with CD8+ T_RM_ cells. CD4+ T_RM_ cells express different cell markers and are affected by different cytokines (43). They have low expression of CD103, and their generation is not interfered by TGF-β, which has a great impact on the generation of CD8+ T_RM_ cell (44, 45). Beyond that, IL-2 and IL-15 were found to affect the differentiation of CD4+ T_eff_ cells in different subsets, respectively (44). Researches on differentiation of CD4+ T_RM_ cells are not as thorough as those on CD8+ T_RM_ cells, and there are still many points to be clarified.

### Maintenance

While persisting in lung tissues, CD8+ and CD4+ T_RM_ cells will construct different structures that contribute to long-term survival. Most CD8+ T_RM_ cells reside in specific niches we refer to as RAMDs, which are constructed by tissue regeneration after tissue damage. These niches are significant for lung CD8+ T_RM_ cells. They may present cytokines that help lung CD8+ T_RM_ cell maintenance. Considering that the recovery of tissue damage takes a long time, the lung CD8+ T_RM_ cells may protect this vulnerable part from secondary infection (35, 36). Unlike CD8+ T_RM_ cells, lung CD4+ T_RM_ cells combine with B cells and other cells to form ectopic lymphoid tissue called inducible bronchus-associated lymphoid tissue (iBALT) that benefits cell survival. In iBALT, CD4+ T_RM_ cells surround B cell follicles, which facilitate rapid interaction with each other and provide a recall response toward potential infection (43, 46). Compared with circulating T_EM_ cells, lung T_RM_ cells displayed different patterns of genes and transcription factors that regulate the expression of cytokine receptors and adhesion molecules, most of which have been mentioned above. Single-cell sequencing found an important transcription factor Notch, which controls the expression of CD103 and the basic metabolic function of lung T_RM_ cells (47). The absence of Notch greatly reduces the population of lung T_RM_ cells. Another study indicated that lung T_RM_ cells were programmed to express IFITM3, which can protect them from secondary infection and improve survival (48). Except for cytokines and surface molecules, M1^hot^ tumor-associated macrophages can also contribute to the maintenance of lung T_RM_ cells in tumor, possibly due to reduction in nutrition competition (49). In comparison with other tissue T_RM_ cells that may persist for a long time or even a lifetime, lung T_RM_ cells gradually disappear 4–5 months after infection. Lung T_RM_ cells that reside in the airway quickly decline due to the harsh environment, where amino acid starvation triggers the integrated stress response, leading to cell apoptosis (50). And those retained in the parenchyma decrease along with the shrink of RMADs. After full regeneration, most of the RAMDs will disappear, and only a minority of lung CD8+ T_RM_ cells may survive in iBALTs (35, 36). In order to compensate for the constant loss, airway T_RM_ cells are replaced primarily by recruitment from lung interstitium (51), and T_RM_ cells in interstitium receive continuous replenishment from circulating T_EM_ cells. T_EM_ cells are recruited and transformed into lung T_RM_ cells under the influence of TGF-β, IL-33, and TNF but antigen-independently. However, T_EM_ cells gradually lose their ability to migrate and convert into lung T_RM_ cells after infection (52). All in all, T_RM_ cells can only provide a short period of protection, which leaves the lung much more susceptible to further infection. However, this may be a designed mechanism for the prevention of pathological immune response.

## Lung T_RM_ Cells Against Infection

**Figure 2:**
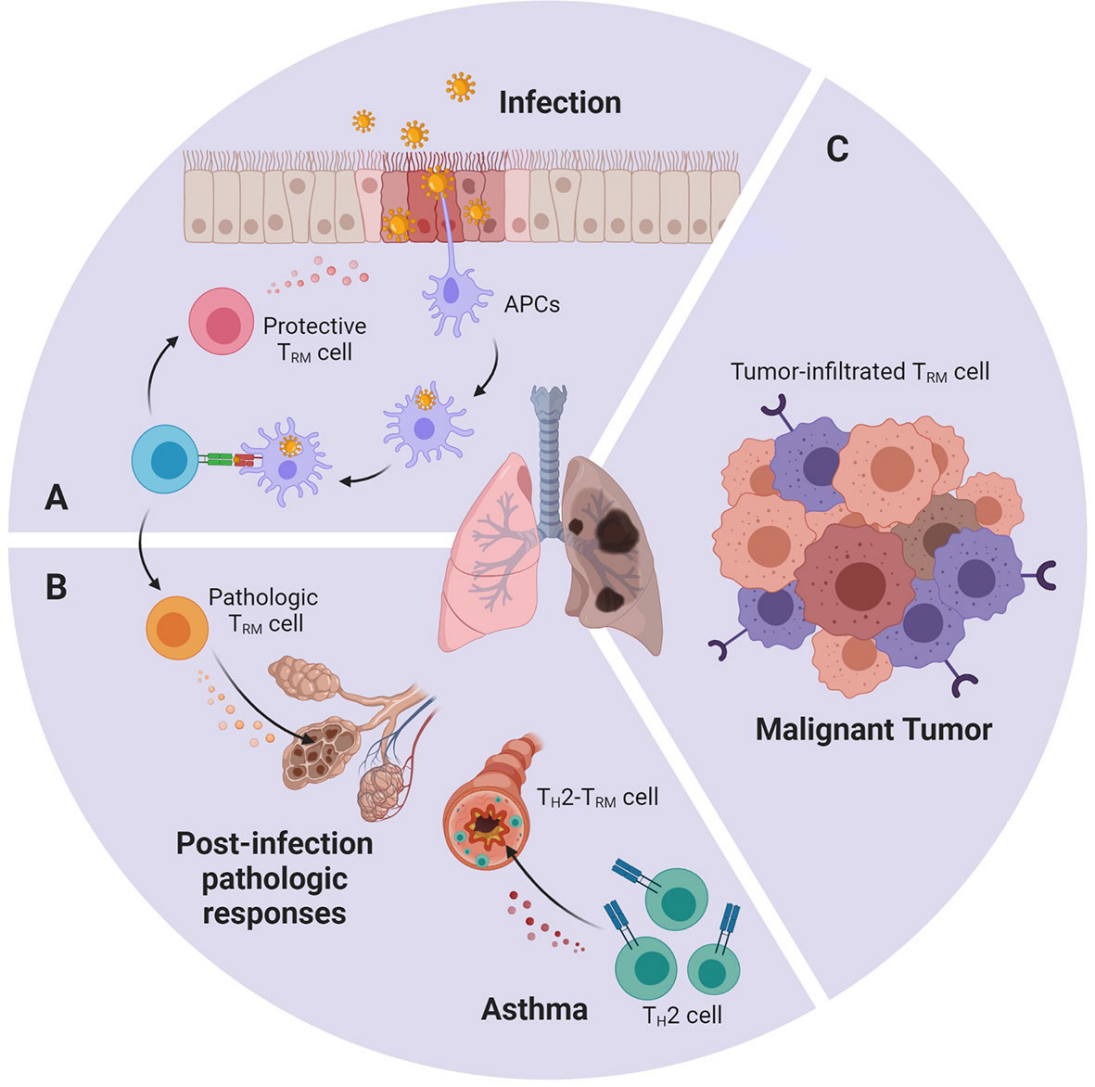
An abstract figure of the role of T_RM_ cells in various lung diseases. Lung T_RM_ cells can: **(A)** rapidly respond towards invasive pathogens during re-infection, **(B)** cause pathologic immune response after overactivated by environmental stimuli or allergen **(C)** infiltrate in lung tumor and express cytotoxic molecules and effector cytokines.

The lungs and respiratory tract, as part of direct access to the outside world, are easily exposed to various pathogens. Common pulmonary pathogens include influenza virus, respiratory syncytial virus (RSV), as well as Streptococcus pneumoniae, Klebsiella pneumoniae, Bordetella pertussis, and Mycobacterium tuberculosis. Under normal circumstances, the first infection caused by these pathogens will not only be cleared by the body’s immune system but also induce memory T cells, some of which settle in the lungs as T_RM_ cells (**Figure 2**).

A large aggregation of studies has shown that the lung is rich in T_RM_ cells specific to a variety of pathogens such as viruses and bacteria. These T_RM_ cells have the potential to mediate immunity against different pathogens and protect the body from re-infection. It has been demonstrated that influenza-specific T_RM_ cells exhibited rapid and robust IFN-γ and TNF-α responses after restimulation *in vitro (* 53, 54). In human RSV challenge model, cells with T_RM_ phenotype can be detected in BALFs, and the higher frequency of RSV-specific CD8+ T_RM_ is related to the decrease in the severity of disease and the viral load (55). CD4+ T_RM_ cells accumulate in the lungs after Bordetella pertussis infection. These cells are pathogen-specific and can secrete IL-17 and/or IFN-γ. A research observed that mice treated with the S1P antagonist Fingolimod (FTY720) to prevent lymphocyte migration into the lungs before initial infection with Bordetella pertussis were significantly more severely affected in the later stages of infection. However, in the case of re-infection, because the tissue-infiltrated T_EM_ cells have partially transformed into T_RM_ cells in the lung, they are not affected by Fingolimod treatment and can still quickly clear the bacillus. At the same time, the adoptive transfer of CD4+ T_RM_ cells from the lungs of mice in convalescence to uninfected mice can protect the latter from pathogens attack (56). All these evidences indicate that T_RM_ cells act as a pivotal role in the rapid response of secondary infection.

However, while T_RM_ cells eliminate invasive pathogens, the released proinflammatory factors such as IFN-γ or perforin and granzymes may damage normal cells, cause lung injury and lead to emphysema or fibrosis, even result in ARDS. Hence, an effective immune response to these infections requires precise immune regulation to eliminate pathogens while protecting the function of normal lung tissue. Many mechanisms exist in the lung to restrict the inflammatory response to acute infection, including inhibitory receptors, immunomodulatory molecules and cells like FOXP3+CD4+ T_reg_ cells (57). Under stable conditions, a large number of T_reg_ cells is reserved in the lung and IL-10 expression is significantly increased after influenza infection (58). In RSV-infected mice, the TCR of T_reg_ cells can specifically recognize the viral epitope-MHC II complex. Immunization of mice with this epitope can reduce clinical manifestations and immunopathology without virus clearance defects (59). In addition, PD-L1 and PD-L2 are expressed in alveolar epithelial cells and are significantly up-regulated to control inflammation in RSV infection (60). However, some studies held that this may limit the formation and development of T_RM_ cells and cause negative effects (61). The detailed mechanisms of lung T_RM_ cell function and immune homeostasis are not yet fully understood, and future improvement in the number and stability of T_RM_ cell population must be carried out on the premise that prevents re-infection of the virus and does not impair the respiratory health of the host.

## Lung T_RM_ Cells in Pathologic Immune Response

As mentioned above, sometimes T_RM_ cells may cease to be the protector and become part of the destructor, and thus attack normal tissue and induce chronic inflammatory diseases (13) (**Figure 2**). After acute influenza infection, antigen deposits in the lung for 2–3 months. In young mice, the persistent presentation of the antigens may induce part of the T_RM_ cells to exhibit exhausted-like phenotype. This phenotype is thought to help maintain lung’s immune balance and prevent damage. If PD-L1 antibody is used to blockade PD-L1 and PD-1 interaction, exhausted-like T_RM_ cells would rejuvenate, express more cytokines, and enhance their heterogeneous protection against infection. But they would also cause pulmonary pathological change and fibrosis (62). In elderly mice, increased expression of TGF-β in the environment led to accumulation of T_RM_ cells in the lungs. However, these T_RM_ cells have low effector activity due to intrinsic defects and fail to enhance the protective function, but can instead lead to chronic inflammation and fibrotic sequela (63). Also, it has been discovered that T_H_2-T_RM_ cells are closely related to asthma (64). They release specific cytokines that recruit eosinophils and maintain mast cells in the airway, which result in the inflammatory response. Using a mouse model exposed to house dust mite (HDM), T_H_2-T_RM_ cells that specifically respond to HDM are identified. These T_H_2-T_RM_ cells are developed from HDM-specific CD4+ T_eff_ cells and are mediated by IL-2 signaling. IL-2 up-regulates chemokine receptors such as CCR4 and CXCR3 that improve migration into the lung, as well as programs related to tissue intention (64). A recently published paper further reports that these T_H_2-T_RM_ cells highly express CD44 and ST2, and can reside in lung tissue and maintain their memory towards allergen for the whole life of a mouse (65). Once re-exposed to allergen, T_H_2-T_RM_ cells robustly proliferate near airways, produce type 2 cytokines, enhance eosinophil activation, and promote peribronchial inflammation. They together with circulating memory T_H_2 cells perform nonredundant function in the induction of asthma (66, 67).

## Lung T_RM_ Cells in Anti-Tumor Immunity

Accumulating evidence suggests that T_RM_ cells are important in anti-tumor immunity (**Figure 2**). It is suggested that a part of the tumor-infiltrating lymphocytes (TILs) isolated from several cancers displays a similar transcriptomic and phenotypic feature with T_RM_ cells. Some refer to it as T_RM_-like TILs (9), but here we still call it “lung tumor T_RM_ cells”, as the consensus in most articles. These lung tumor T_RM_ cells predict a better survival outcome in early-stage non-small-cell lung carcinoma (NSCLC) patients, as well as increased intraepithelial lymphocyte infiltration (68). Single-cell and bulk transcriptomic analysis reveals that lung tumor T_RM_ cells have slightly different transcriptomes compared with other lung T_RM_ cells. They express similar surface marker CD103, CD69, CD49a, and they also up-regulate Notch and Runx3. But lung tumor T_RM_ cells express more cell cycle-related genes, such as CD39, CXCL13, CCL3, and TNFSF4, indicating that they belong to a new subset (22). Comparing samples from different lung cancer patients, the T_RM_ cells of advanced lung cancer are mostly exhausted, while the function of early-stage lung tumor T_RM_ cells is relatively heterogeneous (69). Among them, CD103+CD8+ T_RM_ cells are found to release more cytokines, proliferate faster, and exhibit better anti-tumor performance (70). It is described that CD103 can connect with E-cadherin on tumor cells, which induces cytotoxic granule polarization at the immune synapses (71, 72). CD103 also facilitates T_RM_ cells to reside near tumor tissues (73). In contrast with previous studies, lung tumor T_RM_ cells show the diffuse expression of inhibitory receptors, but do not exhibit the exhausted phenotype. And instead, transcription factor Eomes is found to negatively correlate with T_RM_ cell function (69, 74). Single-cell analysis even discovered a PD-1+TIM-3+IL-7R- T_RM_ cell subset expresses high levels of inhibitory receptors, but remains the ability to proliferate rapidly *in situ* and displays enhanced capacity to express key cytotoxic molecules and effector cytokines (22). Since TIM-3+IL-7R- T_RM_ cells are the major cells expressing PD-1, and CD103+CD8+ T_RM_ cells show positive responses towards anti-PD-1 and anti-PD-L1 monoclonal antibodies, the researchers believe that these cells may be the major subset that reacts in anti-PD-1 therapy (22, 68, 70). In combination with the performance of T_RM_ cells in different stages of lung cancer, it has been speculated that T_eff_ cells were influenced by tumor antigens and cytokines such as TGF-β, up-regulate CD39 and CD103, and converted into CD103+ T_RM_ cells. They exercise their anti-tumor function diligently. If, for one reason or another, the tumor is not eliminated, the local microenvironment as well as the repetitive TCR stimulation may trigger their exhaustion program and they finally become hypofunctional T_RM_ cells (69, 75).

## Vaccination Strategies Inducing Lung T_RM_ Cells

The growing literature that considers T_RM_ cells are indispensable in eliminating infectious pathogens and controlling tumor progression has led to increasing interest in the induction of T_RM_ cells by vaccination for disease treatment and prevention. Compared with circulating T cells or B cells, activated T_RM_ cells are more focused in killing virus-infected cells in target tissues, which help complement neutralizing antibodies and reduce antibodies titer threshold needed to control virus (4, 76, 77).

There are two main strategies to establish T_RM_ cell pool within lung tissues. The first approach applies a one-step method to directly induce antigen-specific lung T_RM_ cells by vaccine vectors (78, 79). For this approach, the route of immunization is very important. Direct intranasal or intrapulmonary route provides better protection compared with commonly used intraperitoneal, intramuscular, or subcutaneous administration route (80, 81). Intranasal administration but not injection of live-attenuated influenza virus has shown the capacity to generate long-term CD4+ and CD8+ T_RM_ cells and provide heterosubtypic protection to nonvaccine influenza strains in mice (82). Intratracheal and intranasal rather than subcutaneous inoculation of Bacille Calmette-Guérin (BCG) also results in generation of T_EM_ and T_RM_ cells in the lung, which remedy the low efficacy of parenteral BCG vaccination to prevent pulmonary TB (83). In a preclinical head and neck cancer model, local T_RM_ cells can be induced and tumor growth can be controlled in mice immunized with the cancer vaccine (STxB-E7) by intranasal route (84). Another approach is a two-step method that combines conventional elicitation of systemic T cell response with the recruitment of these cells into target tissues, which are referred to as “prime and pull” (85). Actually, in a very early stage, scientists have discovered that mucosal boosting with the same vaccine after systemic priming can elicit more CD4+ and CD8+ lung T_RM_ cells compared with only mucosal or systemic vaccination (80). There is also evidence indicates that compared with the original “prime and pull” strategy used in genital tract, the pull step applied in lung disease should use pathogen antigens instead of proinflammatory chemokines. This is because only pathogen antigens can maintain the recruited T cells in airway lumen and persevere immune protection over time (86). Intranasal administration of a novel recombinant anti-TB vaccine (SeV85AB) after subcutaneous immunization with BCG uses this way to provide larger immune protection for lungs than either SeV85AB or BCG alone (87). As opposed to vaccines that directly provide the pathogen antigens like SeV85AB, recent research developed an “antibody-targeted vaccination (ATV)” for the pull step. It connects antigen with antibody that targets lung DC cells, give raise to local antigen presentation, and improve activation of lung T_RM_ cells (88). Pulmonary surfactant-biomimetic liposomes containing stimulator of interferon genes that target alveolar epithelial cells give a new way to recruit CD8+ T_RM_ cells and provide long term wide-spectrum protection (89). These methods may also be used in inducing tumor antigen presentation and lung tumor T_RM_ cell function.

In summary, multiple studies have proved that T_RM_ cells can be induced by vaccination to make a difference in preventing pathogens or controlling tumor growth. However, many problems remained to be solved, for example, how to attract T_eff_ cells into target areas not close to mucosal, and how to maintain long-term lung T_RM_ cells (79). Systemic approaches should also be developed to evaluate the safety and efficiency of these vaccines and prevent overactivation of T_RM_ cells resulting in pathologic immune responses (90).

## Concluding Remarks

It is now obvious that lung T_RM_ cells are an important part of the adaptive immune response within lung tissues. Although we have a rudimentary understanding of lung T_RM_ cells, they remain shrouded in mystery, waiting to be discovered more. While mentioning the migration, activation, differentiation, and maintenance of lung T_RM_ cells, main steps are outlined but there are still huge empties in the details. Do lung T_RM_ cells undergo pre-differentiation in lymph nodes before infection (91)? Which cytokines, transcription factors, and surface molecules are more decisive in the migration, formation, and maintenance of lung CD4+ or CD8+ T_RM_ cells? Are there different subtypes of lung T_RM_ in different lung tissue structures (such as in interstitium and parenchyma)? To answer these questions, more advanced techniques such as single-cell RNA-sequencing that identifies cell-cell interaction and TCR lineage tracking may be used.

A better understanding of these issues will undoubtedly help better manipulate lung T_RM_ cells to prevent or treat disease. Therapy focusing on lung T_RM_ cells in tumor and pathologic immune response is still in a nascent state. Besides direct activation or transmission of tumor-specific T_RM_ cells, currently there are vaccines that activate antiviral lung T_RM_ cells near tumor tissue (92), which reverse the immunosuppressive microenvironment, and may pave the way for later cell therapy. Drugs that prevent lung T_RM_ cell formation or function may also be useful in suppressing the immune response to lung transplantations or preventing lung sequela after respiratory infection in the elderly (63). Of course, T_RM_ cells in the lungs are mostly deemed to fight off lung infections. During the COVID-19 pandemic, lung T_RM_ cells are particularly important in the first line of defense against re-infection of SARS-CoV-2. Actually, influenza viruses have never been conquered, not only because of its versatility, but also because the immune memory only lasts for a short time in lung. To fight them, one possible solution is to improve the “width and depth” of the function of vaccines that induce lung T_RM_ cells. The width refers to the prospect that the same vaccine can induce lung T_RM_ cells that resist a wide range of virus strains in response to virus variability (88). The depth hopes that the induced T_RM_ cells can remain in the lungs for nearly lifelong, enhancing the killing effect and duration of protection of the vaccine (79). More insight and precise manipulation of the fate of lung T_RM_ cells will help to better develop novel immunomodulators to treat lung diseases by T_RM_ cells, and thus to exert the rapid and powerful action in critical illnesses such as COVID-19 pandemic.

## Author Contributions

YQ and YZ contributed to the central idea and coordinated the writing of the manuscript. YQ, YZ, YL, and BL read, discussed, and revised the manuscript. All authors contributed to the article and approved the submitted version.

## Funding

This work was supported by China National Funds for Distinguished Young Scientists (Grant No: 31525008), National Natural Science Foundation of China (Grant Nos: 81830051, 31700775, 31961133011), National Key Research and Development Program of China (Grant No: 2019YFA0906100), and China Postdoctoral Science Foundation (Grant No: 2017M631497).

## Conflict of Interest

BL is a co-founder of Biotheus Inc and the chairman of its scientific advisory board.

The remaining authors declare that the work was conducted in the absence of any commercial or financial relationships that could be construed as a potential conflict of interest.

## References

[1] Medicine JHU Coronavirus Resoure Center (2020). Available at: https://coronavirus.jhu.edu/map.html (Accessed December 1, 2020).

[2] Wiersinga WJ Rhodes A Cheng AC Peacock SJ Prescott HC Pathophysiology, Transmission, Diagnosis, and Treatment of Coronavirus Disease 2019 (COVID-19): A Review. JAMA (2020) 324(8):782–93. 10.1001/jama.2020.12839 32648899

[3] Poland GA Ovsyannikova IG Kennedy RB SARS-CoV-2 immunity: review and applications to phase 3 vaccine candidates. Lancet (2020) 396(10262):1595–606. 10.1016/s0140-6736(20)32137-1 PMC7553736 33065034

[4] Park CO Kupper TS The emerging role of resident memory T cells in protective immunity and inflammatory disease. Nat Med (2015) 21(7):688–97. 10.1038/nm.3883 PMC4640452 26121195

[5] Ibarrondo FJ Fulcher JA Goodman-Meza D Elliott J Hofmann C Hausner MA Rapid Decay of Anti-SARS-CoV-2 Antibodies in Persons with Mild Covid-19. N Engl J Med (2020) 383(11):1085–7. 10.1056/NEJMc2025179 PMC7397184 32706954

[6] Tang F Quan Y Xin ZT Wrammert J Ma MJ Lv H Lack of peripheral memory B cell responses in recovered patients with severe acute respiratory syndrome: a six-year follow-up study. J Immunol (2011) 186(12):7264–8. 10.4049/jimmunol.0903490 21576510

[7] Le Bert N Tan AT Kunasegaran K Tham CYL Hafezi M Chia A SARS-CoV-2-specific T cell immunity in cases of COVID-19 and SARS, and uninfected controls. Nature (2020) 584(7821):457–62. 10.1038/s41586-020-2550-z 32668444

[8] Hogan RJ Usherwood EJ Zhong W Roberts AA Dutton RW Harmsen AG Activated antigen-specific CD8+ T cells persist in the lungs following recovery from respiratory virus infections. J Immunol (2001) 166(3):1813–22. 10.4049/jimmunol.166.3.1813 11160228

[9] Sasson SC Gordon CL Christo SN Klenerman P Mackay LK Local heroes or villains: tissue-resident memory T cells in human health and disease. Cell Mol Immunol (2020) 17(2):113–22. 10.1038/s41423-019-0359-1 PMC7000672 31969685

[10] Channappanavar R Fett C Zhao J Meyerholz DK Perlman S Virus-specific memory CD8 T cells provide substantial protection from lethal severe acute respiratory syndrome coronavirus infection. J Virol (2014) 88(19):11034–44. 10.1128/jvi.01505-14 PMC4178831 25056892

[11] Zhao J Zhao J Mangalam AK Channappanavar R Fett C Meyerholz DK Airway Memory CD4(+) T Cells Mediate Protective Immunity against Emerging Respiratory Coronaviruses. Immunity (2016) 44(6):1379–91. 10.1016/j.immuni.2016.05.006 PMC4917442 27287409

[12] Liao M Liu Y Yuan J Wen Y Xu G Zhao J Single-cell landscape of bronchoalveolar immune cells in patients with COVID-19. Nat Med (2020) 26(6):842–4. 10.1038/s41591-020-0901-9 32398875

[13] Snyder ME Farber DL Human lung tissue resident memory T cells in health and disease. Curr Opin Immunol (2019) 59:101–8. 10.1016/j.coi.2019.05.011 PMC6774897 31265968

[14] Anderson KG Mayer-Barber K Sung H Beura L James BR Taylor JJ Intravascular staining for discrimination of vascular and tissue leukocytes. Nat Protoc (2014) 9(1):209–22. 10.1038/nprot.2014.005 PMC4428344 24385150

[15] Steinert EM Schenkel JM Fraser KA Beura LK Manlove LS Igyártó BZ Quantifying Memory CD8 T Cells Reveals Regionalization of Immunosurveillance. Cell (2015) 161(4):737–49. 10.1016/j.cell.2015.03.031 PMC4426972 25957682

[16] Gebhardt T Wakim LM Eidsmo L Reading PC Heath WR Carbone FR Memory T cells in nonlymphoid tissue that provide enhanced local immunity during infection with herpes simplex virus. Nat Immunol (2009) 10(5):524–30. 10.1038/ni.1718 19305395

[17] Mackay LK Braun A Macleod BL Collins N Tebartz C Bedoui S Cutting edge: CD69 interference with sphingosine-1-phosphate receptor function regulates peripheral T cell retention. J Immunol (2015) 194(5):2059–63. 10.4049/jimmunol.1402256 25624457

[18] Skon CN Lee JY Anderson KG Masopust D Hogquist KA Jameson SC Transcriptional downregulation of S1pr1 is required for the establishment of resident memory CD8+ T cells. Nat Immunol (2013) 14(12):1285–93. 10.1038/ni.2745 PMC3844557 24162775

[19] Kumar BV Ma W Miron M Granot T Guyer RS Carpenter DJ Human Tissue-Resident Memory T Cells Are Defined by Core Transcriptional and Functional Signatures in Lymphoid and Mucosal Sites. Cell Rep (2017) 20(12):2921–34. 10.1016/j.celrep.2017.08.078 PMC5646692 28930685

[20] Mackay CR Marston WL Dudler L Naive and memory T cells show distinct pathways of lymphocyte recirculation. J Exp Med (1990) 171(3):801–17. 10.1084/jem.171.3.801 PMC2187792 2307933

[21] Hadley GA Higgins JM Integrin αEβ7: molecular features and functional significance in the immune system. Adv Exp Med Biol (2014) 819:97–110. 10.1007/978-94-017-9153-3_7 25023170

[22] Clarke J Panwar B Madrigal A Singh D Gujar R Wood O Single-cell transcriptomic analysis of tissue-resident memory T cells in human lung cancer. J Exp Med (2019) 216(9):2128–49. 10.1084/jem.20190249 PMC6719422 31227543

[23] Wein AN McMaster SR Takamura S Dunbar PR Cartwright EK Hayward SL CXCR6 regulates localization of tissue-resident memory CD8 T cells to the airways. J Exp Med (2019) 216(12):2748–62. 10.1084/jem.20181308 PMC6888981 31558615

[24] Mueller SN Mackay LK Tissue-resident memory T cells: local specialists in immune defence. Nat Rev Immunol (2016) 16(2):79–89. 10.1038/nri.2015.3 26688350

[25] Masopust D Soerens AG Tissue-Resident T Cells and Other Resident Leukocytes. Annu Rev Immunol (2019) 37:521–46. 10.1146/annurev-immunol-042617-053214 PMC7175802 30726153

[26] Kim TS Braciale TJ Respiratory dendritic cell subsets differ in their capacity to support the induction of virus-specific cytotoxic CD8+ T cell responses. PLoS One (2009) 4(1):e4204. 10.1371/journal.pone.0004204 19145246 PMC2615220

[27] Mikhak Z Strassner JP Luster AD Lung dendritic cells imprint T cell lung homing and promote lung immunity through the chemokine receptor CCR4. J Exp Med (2013) 210(9):1855–69. 10.1084/jem.20130091 PMC3754856 23960189

[28] Slutter B Pewe LL Kaech SM Harty JT Lung airway-surveilling CXCR3(hi) memory CD8(+) T cells are critical for protection against influenza A virus. Immunity (2013) 39(5):939–48. 10.1016/j.immuni.2013.09.013 PMC3872058 24238342

[29] Jeyanathan M Afkhami S Khera A Mandur T Damjanovic D Yao Y CXCR3 Signaling Is Required for Restricted Homing of Parenteral Tuberculosis Vaccine-Induced T Cells to Both the Lung Parenchyma and Airway. J Immunol (2017) 199(7):2555–69. 10.4049/jimmunol.1700382 28827285

[30] Kohlmeier JE Miller SC Smith J Lu B Gerard C Cookenham T The chemokine receptor CCR5 plays a key role in the early memory CD8+ T cell response to respiratory virus infections. Immunity (2008) 29(1):101–13. 10.1016/j.immuni.2008.05.011 PMC2519120 18617426

[31] Hoft SG Sallin MA Kauffman KD Sakai S Ganusov VV Barber DLJI The rate of CD4 T cell entry into the lungs during Mycobacterium tuberculosis infection is determined by partial and opposing effects of multiple chemokine receptors. Infect Immun (2019) 87(6):e00841–18. 10.1128/IAI.00841-18 PMC6529656 30962399

[32] Richter M Ray SJ Chapman TJ Austin SJ Rebhahn J Mosmann TR Collagen distribution and expression of collagen-binding alpha1beta1 (VLA-1) and alpha2beta1 (VLA-2) integrins on CD4 and CD8 T cells during influenza infection. J Immunol (2007) 178(7):4506–16. 10.4049/jimmunol.178.7.4506 17372009

[33] Laidlaw BJ Zhang N Marshall HD Staron MM Guan T Hu Y CD4+ T cell help guides formation of CD103+ lung-resident memory CD8+ T cells during influenza viral infection. Immunity (2014) 41(4):633–45. 10.1016/j.immuni.2014.09.007 PMC4324721 25308332

[34] D’Souza WN Hedrick SM Cutting edge: latecomer CD8 T cells are imprinted with a unique differentiation program. J Immunol (2006) 177(2):777–81. 10.4049/jimmunol.177.2.777 PMC3137433 16818730

[35] Takamura S Yagi H Hakata Y Motozono C McMaster SR Masumoto T Specific niches for lung-resident memory CD8+ T cells at the site of tissue regeneration enable CD69-independent maintenance. J Exp Med (2016) 213(13):3057–73. 10.1084/jem.20160938 PMC5154946 27815325

[36] Takamura S Persistence in Temporary Lung Niches: A Survival Strategy of Lung-Resident Memory CD8(+) T Cells. Viral Immunol (2017) 30(6):438–50. 10.1089/vim.2017.0016 PMC5512299 28418771

[37] Mackay LK Wynne-Jones E Freestone D Pellicci DG Mielke LA Newman DM T-box Transcription Factors Combine with the Cytokines TGF-β and IL-15 to Control Tissue-Resident Memory T Cell Fate. Immunity (2015) 43(6):1101–11. 10.1016/j.immuni.2015.11.008 26682984

[38] Mackay LK Stock AT Ma JZ Jones CM Kent SJ Mueller SN Long-lived epithelial immunity by tissue-resident memory T (TRM) cells in the absence of persisting local antigen presentation. Proc Natl Acad Sci U S A (2012) 109(18):7037–42. 10.1073/pnas.1202288109 PMC3344960 22509047

[39] McMaster SR Wein AN Dunbar PR Hayward SL Cartwright EK Denning TL Pulmonary antigen encounter regulates the establishment of tissue-resident CD8 memory T cells in the lung airways and parenchyma. Mucosal Immunol (2018) 11(4):1071–8. 10.1038/s41385-018-0003-x PMC6030505 29453412

[40] Behr FM Kragten NAM Wesselink TH Nota B van Lier RAW Amsen D Blimp-1 Rather Than Hobit Drives the Formation of Tissue-Resident Memory CD8(+) T Cells in the Lungs. Front Immunol (2019) 10:400. 10.3389/fimmu.2019.00400 30899267 PMC6416215

[41] Dunbar PR Cartwright EK Wein AN Tsukamoto T Tiger Li ZR Kumar N Pulmonary monocytes interact with effector T cells in the lung tissue to drive TRM differentiation following viral infection. Mucosal Immunol (2020) 13(1):161–71. 10.1038/s41385-019-0224-7 PMC6917844 31723250

[42] Ferreira C Barros L Baptista M Blankenhaus B Barros A Figueiredo-Campos P Type 1 Treg cells promote the generation of CD8(+) tissue-resident memory T cells. Nat Immunol (2020) 21(7):766–76. 10.1038/s41590-020-0674-9 32424367

[43] Schreiner D King CG CD4+ Memory T Cells at Home in the Tissue: Mechanisms for Health and Disease. Front Immunol (2018) 9:2394. 10.3389/fimmu.2018.02394 30386342 PMC6198086

[44] Strutt TM Dhume K Finn CM Hwang JH Castonguay C Swain SL IL-15 supports the generation of protective lung-resident memory CD4 T cells. Mucosal Immunol (2018) 11(3):668–80. 10.1038/mi.2017.101 PMC5975122 29186108

[45] Turner DL Farber DL Mucosal resident memory CD4 T cells in protection and immunopathology. Front Immunol (2014) 5:331. 10.3389/fimmu.2014.00331 25071787 PMC4094908

[46] Hwang JY Randall TD Silva-Sanchez A Inducible Bronchus-Associated Lymphoid Tissue: Taming Inflammation in the Lung. Front Immunol (2016) 7:258. 10.3389/fimmu.2016.00258 27446088 PMC4928648

[47] Hombrink P Helbig C Backer RA Piet B Oja AE Stark R Programs for the persistence, vigilance and control of human CD8(+) lung-resident memory T cells. Nat Immunol (2016) 17(12):1467–78. 10.1038/ni.3589 27776108

[48] Wakim LM Gupta N Mintern JD Villadangos JA Enhanced survival of lung tissue-resident memory CD88^+^ T cells during infection with influenza virus due to selective expression of IFITM3. Nat Immunol (2013) 14(3):238–45. 10.1038/ni.2525 23354485

[49] Garrido-Martin EM Mellows TWP Clarke J Ganesan AP Wood O Cazaly A M1(hot) tumor-associated macrophages boost tissue-resident memory T cells infiltration and survival in human lung cancer. J Immunother Cancer (2020) 8(2):e000778. 10.1136/jitc-2020-000778 32699181 PMC7375465

[50] Hayward SL Scharer CD Cartwright EK Takamura S Li ZT Boss JM Environmental cues regulate epigenetic reprogramming of airway-resident memory CD8(+) T cells. Nat Immunol (2020) 21(3):309–20. 10.1038/s41590-019-0584-x PMC7044042 31953534

[51] Ely KH Cookenham T Roberts AD Woodland DL Memory T cell populations in the lung airways are maintained by continual recruitment. J Immunol (2006) 176(1):537–43. 10.4049/jimmunol.176.1.537 16365448

[52] Slütter B Van Braeckel-Budimir N Abboud G Varga SM Salek-Ardakani S Harty JT Dynamics of influenza-induced lung-resident memory T cells underlie waning heterosubtypic immunity. Sci Immunol (2017) 2(7):eaag2031. 10.1126/sciimmunol.aag2031 28783666 PMC5590757

[53] McMaster SR Wilson JJ Wang H Kohlmeier JE Airway-Resident Memory CD8 T Cells Provide Antigen-Specific Protection against Respiratory Virus Challenge through Rapid IFN-γ Production. J Immunol (2015) 195(1):203–9. 10.4049/jimmunol.1402975 PMC4475417 26026054

[54] Pizzolla A Nguyen TH Sant S Jaffar J Loudovaris T Mannering SI Influenza-specific lung-resident memory T cells are proliferative and polyfunctional and maintain diverse TCR profiles. J Clin Invest (2018) 128(2):721–33. 10.1172/jci96957 PMC5785253 29309047

[55] Jozwik A Habibi MS Paras A Zhu J Guvenel A Dhariwal J RSV-specific airway resident memory CD8+ T cells and differential disease severity after experimental human infection. Nat Commun (2015) 6:10224. 10.1038/ncomms10224 26687547 PMC4703893

[56] Wilk MM Misiak A McManus RM Allen AC Lynch MA Mills KHG Lung CD4 Tissue-Resident Memory T Cells Mediate Adaptive Immunity Induced by Previous Infection of Mice with Bordetella pertussis. J Immunol (2017) 199(1):233–43. 10.4049/jimmunol.1602051 28533445

[57] Yi G Zhao Y Xie F Zhu F Wan Z Wang J Single-cell RNA-seq unveils critical regulators of human FOXP3+ regulatory T cell stability. Sci Bull (2020) 65(13):1114–24. 10.1016/j.scib.2020.01.002 36659163

[58] Bedoya F Cheng GS Leibow A Zakhary N Weissler K Garcia V Viral antigen induces differentiation of Foxp3+ natural regulatory T cells in influenza virus-infected mice. J Immunol (2013) 190(12):6115–25. 10.4049/jimmunol.1203302 PMC3703618 23667113

[59] Liu J Ruckwardt TJ Chen M Nicewonger JD Johnson TR Graham BS Epitope-specific regulatory CD4 T cells reduce virus-induced illness while preserving CD8 T-cell effector function at the site of infection. J Virol (2010) 84(20):10501–9. 10.1128/jvi.00963-10 PMC2950556 20686045

[60] Stanciu LA Bellettato CM Laza-Stanca V Coyle AJ Papi A Johnston SL Expression of programmed death-1 ligand (PD-L) 1, PD-L2, B7-H3, and inducible costimulator ligand on human respiratory tract epithelial cells and regulation by respiratory syncytial virus and type 1 and 2 cytokines. J Infect Dis (2006) 193(3):404–12. 10.1086/499275 16388488

[61] Reagin KL Klonowski KD Incomplete Memories: The Natural Suppression of Tissue-Resident Memory CD8 T Cells in the Lung. Front Immunol (2018) 9:17. 10.3389/fimmu.2018.00017 29403499 PMC5786534

[62] Wang Z Wang S Goplen NP Li C Cheon IS Dai Q PD-1(hi) CD8(+) resident memory T cells balance immunity and fibrotic sequelae. Sci Immunol (2019) 4(36):eaaw1217. 10.1126/sciimmunol.aaw1217 31201259 PMC7458867

[63] Goplen NP Wu Y Son YM Li C Wang Z Cheon IS Tissue-resident CD8(+) T cells drive age-associated chronic lung sequelae after viral pneumonia. Sci Immunol (2020) 5(53):eabc4557. 10.1126/sciimmunol.abc4557 33158975 PMC7970412

[64] Hondowicz BD An D Schenkel JM Kim KS Steach HR Krishnamurty AT Interleukin-2-Dependent Allergen-Specific Tissue-Resident Memory Cells Drive Asthma. Immunity (2016) 44(1):155–66. 10.1016/j.immuni.2015.11.004 PMC4720536 26750312

[65] Bošnjak B Kazemi S Altenburger LM Mokrović G Epstein MM Th2-T(RMs) Maintain Life-Long Allergic Memory in Experimental Asthma in Mice. Front Immunol (2019) 10:840. 10.3389/fimmu.2019.00840 31105692 PMC6493194

[66] Rahimi RA Nepal K Cetinbas M Sadreyev RI Luster AD Distinct functions of tissue-resident and circulating memory Th2 cells in allergic airway disease. J Exp Med (2020) 217(9):e20190865. 10.1084/jem.20190865 32579670 PMC7478729

[67] Turner DL Goldklang M Cvetkovski F Paik D Trischler J Barahona J Biased Generation and In Situ Activation of Lung Tissue-Resident Memory CD4 T Cells in the Pathogenesis of Allergic Asthma. J Immunol (2018) 200(5):1561–9. 10.4049/jimmunol.1700257 PMC5821590 29343554

[68] Djenidi F Adam J Goubar A Durgeau A Meurice G de Montpreville V CD8+CD103+ tumor-infiltrating lymphocytes are tumor-specific tissue-resident memory T cells and a prognostic factor for survival in lung cancer patients. J Immunol (2015) 194(7):3475–86. 10.4049/jimmunol.1402711 25725111

[69] O’Brien SM Klampatsa A Thompson JC Martinez MC Hwang WT Rao AS Function of Human Tumor-Infiltrating Lymphocytes in Early-Stage Non-Small Cell Lung Cancer. Cancer Immunol Res (2019) 7(6):896–909. 10.1158/2326-6066.CIR-18-0713 31053597 PMC6548666

[70] Corgnac S Malenica I Mezquita L Auclin E Voilin E Kacher J CD103+ CD8+ TRM Cells Accumulate in Tumors of Anti-PD-1-Responder Lung Cancer Patients and Are Tumor-Reactive Lymphocytes Enriched with Tc17. Cell Rep Med (2020) 1(7):100127. 10.1016/j.xcrm.2020.100127 33205076 PMC7659589

[71] Le Floc’h A Jalil A Franciszkiewicz K Validire P Vergnon I Mami-Chouaib F Minimal engagement of CD103 on cytotoxic T lymphocytes with an E-cadherin-Fc molecule triggers lytic granule polarization via a phospholipase Cgamma-dependent pathway. Cancer Res (2011) 71(2):328–38. 10.1158/0008-5472.Can-10-2457 21224355

[72] Le Floc’h A Jalil A Vergnon I Le Maux Chansac B Lazar V Bismuth G Alpha E beta 7 integrin interaction with E-cadherin promotes antitumor CTL activity by triggering lytic granule polarization and exocytosis. J Exp Med (2007) 204(3):559–70. 10.1084/jem.20061524 PMC2137907 17325197

[73] Corgnac S Boutet M Kfoury M Naltet C Mami-Chouaib F The Emerging Role of CD8(+) Tissue Resident Memory T (T(RM)) Cells in Antitumor Immunity: A Unique Functional Contribution of the CD103 Integrin. Front Immunol (2018) 9:1904. 10.3389/fimmu.2018.01904 30158938 PMC6104123

[74] Legat A Speiser DE Pircher H Zehn D Fuertes Marraco SA Inhibitory Receptor Expression Depends More Dominantly on Differentiation and Activation than “Exhaustion” of Human CD8 T Cells. Front Immunol (2013) 4:455. 10.3389/fimmu.2013.00455 24391639 PMC3867683

[75] Duhen T Duhen R Montler R Moses J Moudgil T de Miranda NF Co-expression of CD39 and CD103 identifies tumor-reactive CD8 T cells in human solid tumors. Nat Commun (2018) 9(1):2724. 10.1038/s41467-018-05072-0 30006565 PMC6045647

[76] Arunachalam PS Charles TP Joag V Bollimpelli VS Scott MKD Wimmers F T cell-inducing vaccine durably prevents mucosal SHIV infection even with lower neutralizing antibody titers. Nat Med (2020) 26(6):932–40. 10.1038/s41591-020-0858-8 PMC7303014 32393800

[77] Van Braeckel-Budimir N Harty JT Influenza-induced lung Trm: not all memories last forever. Immunol Cell Biol (2017) 95(8):651–5. 10.1038/icb.2017.32 28405016

[78] Zurita ME Wilk MM Carriquiriborde F Bartel E Moreno G Misiak A A Pertussis Outer Membrane Vesicle-Based Vaccine Induces Lung-Resident Memory CD4 T Cells and Protection Against Bordetella pertussis, Including Pertactin Deficient Strains. Front Cell Infect Microbiol (2019) 9:125. 10.3389/fcimb.2019.00125 31106160 PMC6498398

[79] Uddbäck I Cartwright EK Schøller AS Wein AN Hayward SL Lobby J Long-term maintenance of lung resident memory T cells is mediated by persistent antigen. Mucosal Immunol (2021) 14:92–9. 10.1038/s41385-020-0309-3 PMC7726002 32518368

[80] Wang J Thorson L Stokes RW Santosuosso M Huygen K Zganiacz A Single mucosal, but not parenteral, immunization with recombinant adenoviral-based vaccine provides potent protection from pulmonary tuberculosis. J Immunol (2004) 173(10):6357–65. 10.4049/jimmunol.173.10.6357 15528375

[81] Raeven RH Brummelman J Pennings JLA van der Maas L Helm K Tilstra W Molecular and cellular signatures underlying superior immunity against Bordetella pertussis upon pulmonary vaccination. Mucosal Immunol (2018) 11(3):979–93. 10.1038/mi.2017.81 28930286

[82] Zens KD Chen JK Farber DL Vaccine-generated lung tissue-resident memory T cells provide heterosubtypic protection to influenza infection. JCI Insight (2016) 1(10):e85832. 10.1172/jci.insight.85832 PMC4959801 27468427

[83] Perdomo C Zedler U Kuhl AA Lozza L Saikali P Sander LE Mucosal BCG Vaccination Induces Protective Lung-Resident Memory T Cell Populations against Tuberculosis. mBio (2016) 7(6):e01686–16. 10.1128/mBio.01686-16 PMC5120139 27879332

[84] Nizard M Roussel H Diniz MO Karaki S Tran T Voron T Induction of resident memory T cells enhances the efficacy of cancer vaccine. Nat Commun (2017) 8:15221. 10.1038/ncomms15221 28537262 PMC5458068

[85] Shin H Iwasaki A A vaccine strategy that protects against genital herpes by establishing local memory T cells. Nature (2012) 491(7424):463–7. 10.1038/nature11522 PMC3499630 23075848

[86] Santosuosso M McCormick S Roediger E Zhang X Zganiacz A Lichty BD Mucosal luminal manipulation of T cell geography switches on protective efficacy by otherwise ineffective parenteral genetic immunization. J Immunol (2007) 178(4):2387–95. 10.4049/jimmunol.178.4.2387 17277145

[87] Hu Z Wong KW Zhao HM Wen HL Ji P Ma H Sendai Virus Mucosal Vaccination Establishes Lung-Resident Memory CD8 T Cell Immunity and Boosts BCG-Primed Protection against TB in Mice. Mol Ther (2017) 25(5):1222–33. 10.1016/j.ymthe.2017.02.018 PMC5417795 28342639

[88] Wakim LM Smith J Caminschi I Lahoud MH Villadangos JA Antibody-targeted vaccination to lung dendritic cells generates tissue-resident memory CD8 T cells that are highly protective against influenza virus infection. Mucosal Immunol (2015) 8(5):1060–71. 10.1038/mi.2014.133 25586557

[89] Wang J Li P Yu Y Fu Y Jiang H Lu M Pulmonary surfactant-biomimetic nanoparticles potentiate heterosubtypic influenza immunity. Science (New York NY) (2020) 367(6480):eaau0810. 10.1126/science.aau0810 PMC7432993 32079747

[90] Muruganandah V Sathkumara HD Pai S Rush CM Brosch R Waardenberg AJ A systematic approach to simultaneously evaluate safety, immunogenicity, and efficacy of novel tuberculosis vaccination strategies. Sci Adv (2020) 6(10):eaaz1767. 10.1126/sciadv.aaz1767 32181361 PMC7056300

[91] Mani V Bromley SK Aijo T Mora-Buch R Carrizosa E Warner RD Migratory DCs activate TGF-beta to precondition naive CD8(+) T cells for tissue-resident memory fate. Science (New York NY) (2019) 366(6462):eaav5728. 10.1126/science.aav5728 PMC6939608 31601741

[92] Rosato PC Wijeyesinghe S Stolley JM Nelson CE Davis RL Manlove LS Virus-specific memory T cells populate tumors and can be repurposed for tumor immunotherapy. Nat Commun (2019) 10(1):567. 10.1038/s41467-019-08534-1 30718505 PMC6362136

